# Identification of Phlogacantholide C as a Novel ADAM10 Enhancer from Traditional Chinese Medicinal Plants

**DOI:** 10.3390/medicines3040030

**Published:** 2016-12-05

**Authors:** Myriam Meineck, Florian Schuck, Sara Abdelfatah, Thomas Efferth, Kristina Endres

**Affiliations:** 1 Clinic of Psychiatry and Psychotherapy, University Medical Center of the Johannes Gutenberg-University Mainz, 55131 Mainz, Germany; mmeineck@students.uni-mainz.de (M.M.); florian.schuck@unimedizin-mainz.de (F.S.); 2 Institute of Pharmacy, Johannes Gutenberg-University Mainz, 55099 Mainz, Germany; saabdelf@uni-mainz.de (S.A.); efferth@uni-mainz.de (T.E.)

**Keywords:** ADAM10, Amyloid precursor protein, Alzheimer’s disease, Norkurarinol, Phlogacantholide C, *Phlogacanthus curviflorus*, *Sophora flavescens*

## Abstract

**Background:** Alzheimer’s disease is one of the most prevalent dementias in the elderly population with increasing numbers of patients. One pivotal hallmark of this disorder is the deposition of protein aggregates stemming from neurotoxic amyloid-beta peptides. Synthesis of those peptides has been efficiently prevented in AD model mice by activation of an enzyme called alpha-secretase. Therefore, drugs with the capability to increase the expression of this enzyme, named ADAM10, have been suggested as a valuable therapeutic medication. **Methods:** We investigated 69 substances from a drug library derived from traditional Chinese medicine by luciferase reporter assay in human neuronal cells for their potential to selectively induce alpha-secretase expression. Western blot analysis was used to confirm results on the protein level. **Results:** Ten of the 69 investigated compounds led to induction of ADAM10 transcriptional activity while BACE-1 (beta-site APP cleaving enzyme 1) and APP (amyloid precursor protein) expression were not induced. Two of them—Norkurarinol and Phlogacantholide C—showed substantial elevation of ADAM10 protein levels and Phlogacantholide C also increased secretion of the ADAM10-derived cleavage product APPs-alpha. **Conclusion:** Phlogacantholide C represents a novel ADAM10 gene expression enhancer from traditional Chinese medicinal herbs that may lay the groundwork for evolving potential novel therapeutics in Alzheimer’s disease.

## 1. Introduction

As life expectancy in most civilizations has tremendously increased during the last 100 years, diseases of the advanced life period have come into the focus of research. Alzheimer’s disease is a slowly progressing neurodegenerative disease which is clinically characterized by cognitive decline and changes of personality [1]. An estimated 40 million people worldwide suffer from dementia, with Alzheimer’s disease being the most prevalent, at least in the elderly [2]. The origin of the disease still remains enigmatic despite the few genetically based cases (1%–3% of all cases [3]). This, as a consequence, hampers the development of efficient targeted medication. One molecular hallmark of the disease is the deposition of neurotoxic amyloid-beta peptides that derive from the proteolytic processing of the amyloid precursor protein (APP [4]) by beta-secretase BACE-1 and gamma-secretase [5,6]. However, involvement of these peptides has been controversially discussed in the field, and clinical trials which solely focused on preventing synthesis of the peptides have failed so far [7]. Targeting the alpha-secretase ADAM10 (a disintegrin and metalloproteinase 10) in this regard represents an attractive alternative: it not only prevents amyloid-beta generation by cutting within the respective peptide stretch but it also liberates the proteolysis fragment APPs-alpha [8]. The latter has been assigned beneficial effects for neuronal cells such as promoting outgrowth of neurites and preventing neuronal death as well as mitigating synaptic and cognitive deficits in AD (Alzheimer’s disease) mouse models [9,10,11,12].

Plant extracts have been used for more than 2000 years by indigenous populations for treating disorders including forms of dementia and memory impairment (as reviewed in [13]). Huge parts of the world’s population still rely on traditional medicine (TM) for their primary health care [14]: for example, in Korea as well as China 15.26% and 12.63% TM doctors practice in hospitals and clinics [15]. Western medicine shows increasing interest in isolating novel lead compounds from such traditionally used medications (reviewed in [16]). A recent example for a potential anti-AD therapeutic drug is given by caffeoylquinic acid, found, for example, in coffee beans, which has been shown to be protective against amyloid-beta–induced cytotoxicity and to reduce beta-sheet formation of amyloid-beta peptides in neuroblastoma cell lines [17,18]. In 2015 we described the identification of alpha-viniferin from the stem bark of *Caragana sinica* as an ADAM10 gene expression enhancer from a bank of traditional Korean medicinal plant extracts [19]. As part of the ongoing search for biologically active compounds from traditional Chinese medicine, we here report the investigation of a 69-compound-containing library which revealed the anti-amyloidogenic activity of Phlogacantholide C from *Phlogacanthus curviflorus* [20].

*Phlogacanthus curviflorus* (Wall.) Nees (Acanthaceae) is a large branched shrub which grows in Yunnan Province of China as well as, e.g., in Vietnam and India [21], and reaches up to 3 to 4 m. Oppositely arranged elliptic leaves are 8 to 10 in long. The tube-like reddish flowers are borne in upright spikes at the end of the branches. In North India, boiled leaf juice is used to cure cough and fever, and flowers are eaten raw or fried or used as a spice [22]. Moreover, it is used in the postpartum herbal bath of the Mien population in Northern Thailand, probably due to its antioxidant properties [23].

## 2. Materials and Methods

### 2.1. Plant Material

Medicinal plants were collected or purchased in China [24], mainly from Yunnan province (600 to 700 m above sea level). The botanical identification was done as described before [24] and voucher specimens deposited at the herbarium of the State Key Laboratory of Phytochemistry and Plant Resources in West China, Kunming, Institute of Botany, Chinese Academy of Sciences, Kunming, P.R. China. The finely ground plant material was successively extracted with solvents of increasing polarity (petroleum ether (or *n*-hexane), ethyl acetate, and methanol) as described before [24,25] and finally solved in DMSO. For detailed information about the tested substances see [25].

### 2.2. Cell Culture

SH-SY5Y human neuroblastoma cells were maintained at humidified air (95%), 5% CO_2_ and 37 °C. Cultivation was performed using DMEM/F12 (Gibco, Fisher Scientific GmbH, Schwerte, Germany) supplemented with 10% fetal calf serum and 1% Glutamine. Cells were passaged twice a week 1:2–1:4.

### 2.3. Cytotoxicity Test

Potential cytotoxic effects were assessed by using the Cell Titer Glo-Assay (Promega, Mannheim, Germany) in 96-well formats (white plate with glass bottom). Initial drug concentration was 0.1% vol/vol in 50 μL culture medium and an incubation period of 48 h was tested. After 48 h of incubation, 50 μL of assay reagent were added and the ATP content (as a surrogate parameter for viability) measured using the Fluostar Optima luminometer (BMG Labtech, Cary, NC, USA). Ten measurements were taken from each well (interval time 0.5 s) and means calculated. Concentrations were adjusted if necessary (toxic or pro-proliferative effects) to obtain non-toxic dosages.

### 2.4. Transfection and Promoter Assays

A transient retro-cotransfection of two luciferase reporter plasmids (depending on ADAM10 or BACE-1 promoter activity) was conducted in SH-SY5Y cells as described previously [26]. In brief, to each well of a 96-well plate 20 μL Opti-MEM containing 100 ng of each luciferase encoding vector were added and incubated for 20 min at room temperature. Subsequently, 20 μL of Opti-MEM containing 0.3 μL transfection reagent (Lipofectamine 2000, Fisher Scientific GmbH, Schwerte, Germany) were added to each well and incubated for 45 min at room temperature. 1.5 × 10^5^ cells per cm^2^ surface area of the dish were seeded. After 5 h of incubation, medium was exchanged to full cultivation medium containing DMSO (control) or the herbal drug in the indicated concentration and transfected cells were cultivated for 48 h. Cells were lysed in 20 μL passive lysis buffer (Promega), lysis promoted by freezing overnight at −20 °C and Renilla and firefly luciferase activity assessed using a reporter assay kit (Dual-Luciferase Reporter Assay, Promega) and the Fluostar Optima luminometer (BMG). The ratio of ADAM10-promoter activity (firefly luciferase) to BACE-1-promoter activity (Renilla luciferase) was calculated and the transcriptional activity of control cells was set to 100%. Hits were considered as follows: values > mean + SD (130%) of control-treated cells.

For the APP-promoter assay the procedure was comparable using a singular reporter vector based on the pGL4.76 plasmid (Promega) which has been describe before [26].

### 2.5. Western Blotting

For analysis of the drug-induced effect on ADAM10 expression and non-amyloidogenic APP-processing, cells were seeded on 24-well plates (1.3 × 10^5^ cells per cm^2^ surface area) and incubated with the indicated drugs or DMSO for 48 h. Secretion medium was collected for the last 5 h following a medium exchange to FCS-free medium containing the respective drug or DMSO as a solvent. Secreted proteins were precipitated by trichloroacetic acid as described before (e.g., [27]). Cell lysates were prepared using Nu-PAGE-buffer (Fisher Scientific GmbH, Schwerte, Germany) supplemented with 10 vol % 1 M DTT. Samples were subjected to 8% SDS polyacrylamide gels and proteins separated at 70–120 V. Subsequently, proteins were transferred via tank blot procedure (2 h, 100 V, BioRad apparatus, Hercules, CA, USA) onto nitrocellulose membranes (blocked with 0.2% I-Block solution (Fisher Scientific GmbH, Schwerte, Germany) and incubated with the appropriate primary antibodies followed by secondary horse radish peroxidase-coupled antibodies (Fisher Scientific GmbH, Schwerte, Germany). Detection of signals was performed by CCD camera (Raytest, Straubenhardt, Germany) and densitometric analysis by Aida 3.5 (Raytest). Actin served as a loading control.

## 3. Results and Discussions

The collection and botanical identification of medicinal plants mainly from the Yunnan Province, China, have been described [24]. The bioactivity-guided isolation of phytochemicals by chromatographic methods was performed as previously described [28,29]. The chemical structures were elucidated by spectrometric methods and crystal structure analysis [30].

### 3.1. Results for Toxicity Assay

**Figure 1:**
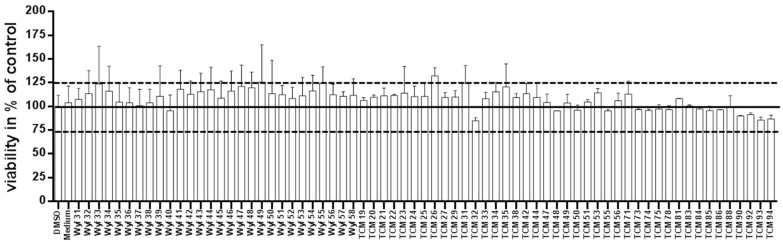
Toxicity assay of tested substances. SH-SY5Y cells were incubated for 48 h with 0.1% vol/vol substance and viability was assessed using the Cell Titer Glo assay. DMSO (solvent) and pure culture medium served as controls. Values ± SD were collected from at least two independent experiments. Dashed lines indicate the maximum tolerated proliferative or toxic effect. Concentration of the following substances had to be adjusted by dilution with DMSO as indicated to obtain reliable viability measurements: TCM48: 0.05%; TCM19-22, 51, 54, 81: 0.01% (only the viability measurements for the adjusted concentrations are shown in the figure).

Starting from 0.1% vol/vol, 90% of the tested TCM (traditional Chinese medicine)-derived substances revealed no toxic effect on the human neuroblastoma cell line SH-SY5Y (61 out of 69, Figure 1). Only for a minority the concentration had to be reduced (only results from adjusted concentrations are presented, data from initial measurements with higher concentrations are not shown). For TCM19 (Sophoraflavon G) and TCM22 (Norkurarinol), for example, a further dilution to 0.01% evoked no viability-decreasing results. Sophoraflavanone G (5,7,2′,4′-tetrahydroxy-8-lavandulylflavanone), a close relative to Sophoraflavon G, has been referred to as a phytochemical with an intense antibacterial activity which might be due to its capability to reduce the fluidity of the outer and inner layers of membranes [31]. This was also assumed for Sophoraflavon G and thereby might explain the toxic effect on the human cells used in our study occurring at higher dosage. Norkurarinol, also a flavonoid extracted from *Sophora flavescens*, has been shown to exert cytotoxic effects in cancer cells, probably due to its tyrosinase inhibitor properties [32].

### 3.2. Results for Dual Promoter Assay

**Figure 2:**
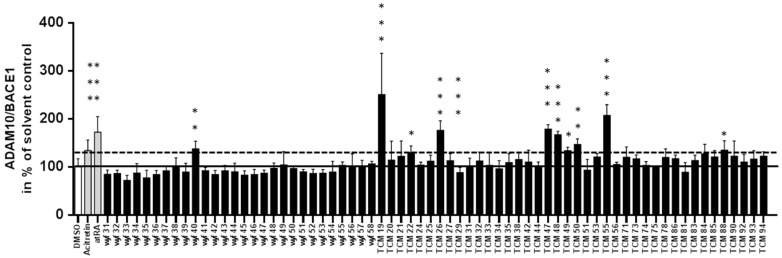
Influence of tested substances on ADAM10/BACE-1 promoter activity ratio. Cells were incubated for 48 h with substances according to results from toxicity assay (see Figure 1). DMSO (solvent) and known activators of ADAM10 promoter activity (Acitretin and all-trans retinoic acid, both 1 μM, [34]) served as controls. The dashed line indicates the minimal effect size expected for a “hit” (values > mean + SD (130%) of control-treated cells). Values ± SD were collected from three independent experiments (statistical analysis: one-way ANOVA with Bonferroni’s multiple comparison test; ***, *p* < 0.001; **, *p* < 0.01; *, *p* < 0.05).

TCM-derived substances were administered to the dual promoter assay for assessing the potential anti-amyloidogenic property. Ten out of the 69 tested substances revealed an ADAM10 promoter–inducing effect and none indicated an induction of BACE-1 promoter activity (Figure 2). The effect size was comparable for some substances to already known ADAM10 expression enhancers such as Acitretin or atRA [33,34], Wyf40, TCM22, 26, 47, 48, 49, 50, 88, while others displayed rather high induction rates (TCM19: 250% of control; TCM55: 207% of control). To make sure that none of the observed luciferase measurements was due to a direct effect on the enzymatic reaction, this was tested separately by an in vitro incubation of luciferase-containing cell lysate with the respective substance (data not shown).

An ADAM10-enhancing effect with regard to Alzheimer’s disease should not be paralleled by an increase in the substrate expression itself. For instance, a higher gene dosage of APP alone is sufficient in Trisomy 21 patients to result in Alzheimer-type dementia [35]. Therefore, we tested if the nine candidates identified by the dual promoter assay inherit the inducing potential on the human APP promoter (see Table 1; TCM26 was not included due to lack of sufficient material for further analyses). None of the selected substances displayed a drastic induction or reduction of the APP transcriptional activity. APP promoter activity ranged from 117% to 61% of the control, but effects did not reach statistical relevance in comparison to solvent-treated cells.

### 3.3. Results for ADAM10 Expression and Enzymatic Activity

**Figure 3:**
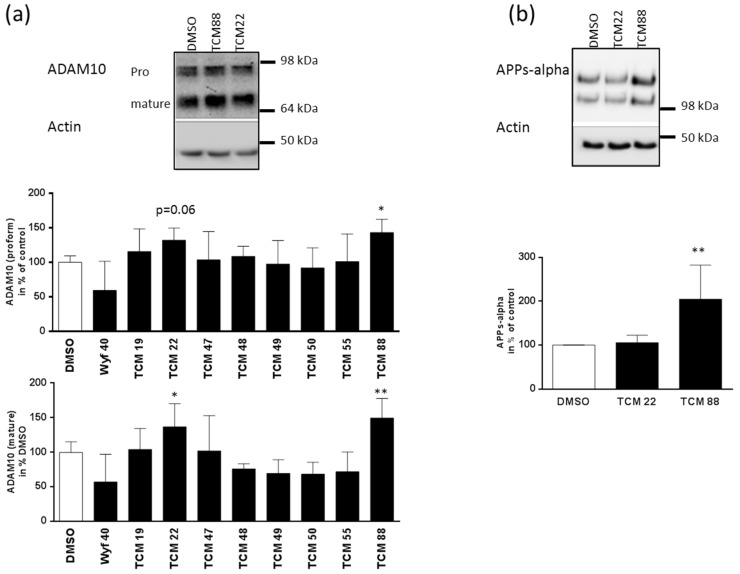
Influence of candidate substances on ADAM10 expression and APPs-alpha secretion. (**a**) Expression of ADAM10. Cells were incubated for 48 h with substances according to results from toxicity assay in FCS-containing medium (see Figure 1). DMSO (solvent) served as control. Twenty percent of cell lysates were subjected to Western blotting and ADAM10 was detected with Calbiochem antibody (dilution 1:1000). Actin served as a loading control (antibody (Sigma-Aldrich, Darmstadt, Germany) diluted 1:1000). Pro- and mature forms of the enzyme were measured, normalized to Actin and depicted in % of DMSO control-treated cells. Values ± SD were collected from three independent experiments. An exemplary blot for analysis of DMSO, TCM22 and 88 is shown. (**b**) Effect of TCM22 and 88 on APP processing. For secretion experiments, cells were incubated for the last 5 h in FCS-free medium supplemented with the candidate substances. The whole amount of precipitated proteins from cell supernatant was used for detection of APPs-alpha (antibody 6E10 (BioLegend, Fell, Germany)) and 20% of the cell lysate for detection of Actin to ascertain comparable amounts of cells in the experimental setting. Values obtained for APPs-alpha were normalized to Actin measurements and presented in % of solvent control (*n* = 6, four independent experiments). Statistical analysis: one-way ANOVA; **, *p* < 0.01; *, *p* < 0.05.

Substance characterization based on reporter gene assays bears the problem of investigating an isolated DNA sequence without the genomic environment. Additionally, translation and stability of the protein products are not integrated in those investigations. We therefore analyzed the outcome of cultivating SH-SY5Y cells with the candidates from the dual promoter assay on the endogenous ADAM10 protein level (Figure 3A). For substances TCM22 (Norkurarinol) and 88 (Phlogacantholide C), the effect observed on the promoter construct was substantiated within the protein quantitation while those of the seven other compounds could not be substantiated: for TCM22 the immature proform of the enzyme was elevated to 132%, and the mature form to 137%. For TCM88 an induction to 143% for the proform and to 150% for the mature form was observed. For TCM88, additionally, an increase of APPs-alpha secretion of 200% as compared to solvent-treated cells was observed (Figure 3B). This indicates that not only the amount but also the activity of ADAM10 has been induced by Phlogacantholide C. We can only speculate why TCM22 failed to induce APPs-alpha secretion despite its effect on the ADAM10 amount: although we did not observe an influence on the APP promoter in our reporter gene assay, Norkurarinol might lead to a reduced APP protein amount at the cell surface or the newly built ADAM10 might be dislocated and therefore unable to cleave APP.

## 4. Conclusions

We identified Phlogacantholide C from *Phlogacanthus curviflorus* [20,36] as a new ADAM10 gene expression enhancer from a bank containing 69 substances derived from traditional Chinese medicinal herbs. *Phlogacanthus curviflorus* is used in traditional medicine as an anti-malarian drug [36] or in the context of curing or preventing inflammatory events [22,23]. However, no biological or pharmaceutical investigation regarding the isolated diterpene lactone glucoside has yet been reported to our knowledge.

## Figures and Tables

**Table 1:** Candidate substances selected from dual promoter assay.

Substance Code	Substance	Formula	Plant	Concentration (mg/mL)	Effect on APP Promoter (MW ± SD)
Wyf40	Cynaropicrin	C_19_H_22_O_6_ 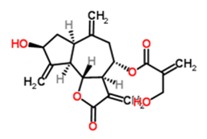	*Saussurea deltoidea*	2	93.13 ± 24.96
TCM19	Sophoraflavon G	C_25_H_28_O_6_ 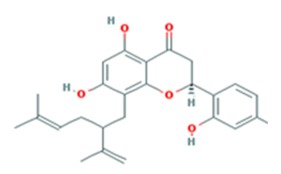	*Sophora flavescens*, *Sophora pachycarpa*, and *Sophora exigua*	2	115.3 ± 58.45
TCM22	Norkurarinol	C_25_H_30_O_7_ 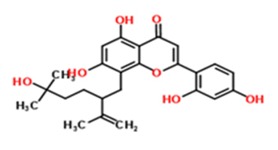	*Sophora flavescens*	2	84.50 ± 47.86
TCM47	5-Methoxy-3-methyl-9*H*-carbazol-2-ol	C_14_H_13_NO_2_ 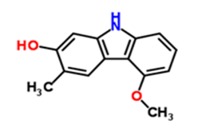	*Glycosmis pentaphylla*	12	86.88 ± 36.46
TCM48	7-methoxyglycomaurin	C_18_H_17_NO_2_ 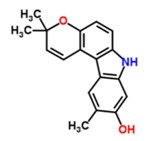	*Glycosmis rupestris*	12	61.38 ± 25.81
TCM49	glybomine B	C_19_H_21_NO_2_ 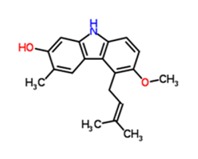	*Glycosmis arborea*	12	68.57 ± 34.91
TCM50	(2*E*)-2-Methyl-4-[7-(3-methyl-2-buten-1-yl)-1*H*-indol-3-yl]-2-buten-1-ol	C_18_H_23_NO 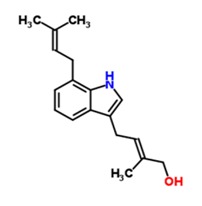		12	117.8 ± 53.42
TCM55	(4*R*,4a*S*,8a*R*,10*R*,10a*R*,12*S*,13*S*,14b*S*)-4-methyl-12-((methylthio)methyl)decahydro-1*H*,8a*H*,10*H*,11*H*-4,14b,10-(epiethane[1,1,2]triyl)-10a,13-ethanoisochromeno[4,3-g]oxazolo[3,2-a]azocin-11-one	C_23_H_33_NO_3_S 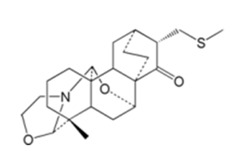	*Spiraea japonica*	4	96.75 ± 53.34
TCM88	Phlogacantholide C	C_20_H_28_O_4_ 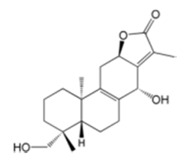	*Pholgacanthus curviflorus*	2	80.25 ± 31.15
